# Antimicrobial susceptibility profiling of caprine clinical mastitis pathogens in Punjab, India

**DOI:** 10.1097/MS9.0000000000003522

**Published:** 2025-06-23

**Authors:** Priyanka Choudhary, Sunil Punia, Narinder Singh Sharma

**Affiliations:** a Department of Veterinary Microbiology, College of Veterinary Science, Guru Angad Dev Veterinary and Animal Sciences University Rampura Phul, Bathinda, Punjab, India; b Department of Veterinary Medicine, College of Veterinary Science, Guru Angad Dev Veterinary and Animal Sciences University, Rampura Phul, Bathinda, Punjab, India

**Keywords:** antibiotic, antimicrobial resistance, goat, mastitis, susceptibility

## Abstract

**Background:**

Goats can serve as an efficient animal model to study antimicrobial resistance. The present study was undertaken with an aim to assess the status of antibiotic resistance associated with caprine mastitis in the Bathinda district of Punjab.

**Methods:**

Milk samples were collected aseptically from the cases of clinical mastitis in goats followed by bacterial isolation and antibiotic susceptibility testing for amikacin, amoxicillin, amoxyclav, ampicillin, ampicillin-sulbactam, cefoperazone, cefoperazone-tazobactam, ceftizoxime, ceftriaxone, ceftriaxone-sulbactam, ceftriaxone-tazobactam, ciprofloxacin, colistin, doxycycline, enrofloxacin, erythromycin, gentamicin, moxifloxacin, ofloxacin, oxytetracycline, penicillin-G, streptomycin and tetracycline.

**Results:**

The antimicrobial susceptibility testing revealed resistance to multiple antibiotics including the macrolide (100%) and polymyxin (100%) group followed by the penicillins (88.89%), tetracyclines (43.75%), cephalosporins (28.57%), aminoglycosides (25.93%), and quinolones (13.33%). The resistance in penicillin (28.57%) and cephalosporin (20%) combinations was comparatively lower as compared to their individual use. Among the bacterial isolates, *Escherichia coli* (21.42%) were multidrug resistant to more than five antibiotics tested, whereas *Bacillus* species (21.42%) showed resistance to three to five antibiotics and; *Staphylococcus* spp. (35.71%), *Streptococcus* spp. (7.14%) and *Corynebacterium* spp. (14.28%) were resistant to less than three antibiotics tested.

**Conclusion:**

As the antimicrobial susceptibility was found to vary among the goats suffering from mastitis as well as the bacteria involved, the antimicrobial susceptibility testing prior to treatment initiation would be crucial in limiting the development of resistance in goats and potentially in human beings as well.

**Statement of novelty:**

The antimicrobial susceptibility profiling in caprine mastitis revealed the multidrug resistant bacterial isolates.

**HIGHLIGHTS**

- The antimicrobial susceptibility testing of milk samples from caprine clinical mastitis revealed resistance to multiple antibiotics including a very high resistance against antibiotics belonging to the macrolide and polymyxin group followed by the penicillins and tetracyclines; resistance against the cephalosporins, aminoglycosides and quinolones being quite lower.
- The resistance in penicillin and cephalosporin combinations was comparatively lower as compared to their individual use.
- The microbiological testing of the milk samples of goat suffering from clinical mastitis revealed the presence of *Bacillus* spp. (21.42%), *Corynebacterium* spp. (14.28%), *Escherichia coli* (21.42%), *Staphylococcus* spp. (35.71%) and *Streptococcus* spp. (7.14%). Among these bacterial isolates, *Escherichia coli* were multidrug resistant to more than five antibiotics tested, whereas *Bacillus* species showed resistance to three to five antibiotics and; *Staphylococcus* spp., *Streptococcus* spp. and *Corynebacterium* spp. were resistant to less than three antibiotics tested.

## Introduction

Goat farming has been progressively flourishing in the rural households throughout India, including Punjab. This may be attributable to their high adaptability in adverse environments^[1]^ and minimal maintenance cost. As per the data-based predictions on livestock populations from the year 2000 to 2050, goat and sheep would constitute the highest population among the livestock, reaching about 1.7 to 2.7 billion animals^[2]^. However, the limited understanding of the diseases and lack of veterinary services hinder the efficient and cost-effective goat farming^[3]^. A number of infectious diseases affect goat farming with the most common one being the mastitis^[4]^. Mastitis is the inflammation of the mammary glands which results due to infection by pathogenic microorganisms including bacteria, fungi and viruses. Infectious agents, host resistance and environment are the three major factors associated with mastitis^[5]^. Bacteria inhabiting the udder and skin of teats, such as the *Staphylococcus aureus* and *Streptococcus agalactiae* are responsible for contagious mastitis which is transmitted from one goat to another. The environmental pathogens like *E. coli, Streptococcus uberis* and *Klebsiella* spp. which are usually present on bedding, manure, soil and feed, cause environmental mastitis. Timely detection and treatment of mastitis is imperative for the mitigation of disease and the consequential economic losses.

Antibiotics, primarily the penicillin, oxytetracycline, florfenicol, gentamicin, ampicillin, amoxicillin and tylosin, are used for the treatment of infectious diseases in goats^[6]^. However, the antibiotic resistance may develop on their exposure to the commensal or pathogenic microorganisms^[7]^. Antimicrobial resistance is a worldwide threat which has severely affected the animal and human health besides impacting the economic sector as evident by the increased morbidity and mortality in goats^[8]^ because of the failure to treat the bacterial infections. The bacteria in livestock including the goats, can serve as reservoir of the antibiotic resistance genes like ESBL in members of the *Enterobacteriaceae* which confer resistance to extended-spectrum cephalosporins including cefotaxime, ceftazidime, ceftiofur and ceftriaxone and have also been involved in the resistance to fluoroquinolones, aminoglycosides and trimethoprim-sulfamethoxazole combination^[9]^. The previous studies have reported the antimicrobial resistance in the mastitis-causing pathogens due to usage of the antibiotics for long-term^[10]^ or irrationally and incompletely^[11]^.

Besides, the antibiotic resistant genes in goats also pose a risk for the human health because of animal-human interactions^[12]^. Goats can serve as an efficient animal model to study antimicrobial resistance through the investigation of its emergence, transmission and the mechanisms involved. They can enable the tracking of the development and spread of antimicrobial resistance in animals and potentially in human beings. Hence, at par with the studies done on bovine mastitis in India, it is imperative to study the prevalence of AMR in small ruminants such as goats as well, so that policy decisions regarding antimicrobial usage may be undertaken. Therefore, the present study was undertaken with an aim to assess the status of antibiotic resistance associated with caprine mastitis in the Bathinda district of Punjab.

### Materials and methods

#### Sample collection:

A total of 17 aseptically collected milk samples from the cases of clinical mastitis in goats in the Bathinda district of Punjab referred from the Teaching Veterinary Clinical Complex, College of Veterinary Science, Rampura Phul during 2022-2025 were used in the study.

#### Microbiological investigation:

The bacterial isolation was done as per the routine microbiological procedures. Milk samples were inoculated on Brain Heart Infusion agar and incubated at 37°C for 24-48 hours, followed by sub-culturing of individual colonies for purification. Various genera of the pure bacterial isolates were identified based on their cultural and staining characteristics and evaluation of their biochemical profile as per the standard protocols described by Cruickshank *et al*^[13]^, Carter^[14]^ and Forbes *et al*^[15]^.

#### Antimicrobial susceptibility testing:

Pure isolated colonies were inoculated into sterile Brain Heart Infusion broth and incubated at 37°C for 5 to 6 hours till the turbidity matched with the standard of 0.5 McFarland solutions (0.08-0.13 OD at 620 nm). This was followed by antibiotic susceptibility testing by agar disc diffusion method on Mueller-Hinton Agar plates as per the Clinical and Laboratory Standards Institute (CLSI) guidelines (CLSI, 2020). The commercially available antibiotic discs (HiMedia) used along with their concentrations included Amikacin (30 mcg), Amoxicillin (10 mcg), Amoxyclav (30 mcg), Ampicillin (10 mcg), Ampicillin-Sulbactam (10/10 mcg), Cefoperazone (75 mcg), Cefoperazone-Tazobactam (75/10 mcg), Ceftizoxime (30 mcg), Ceftriaxone (30 mcg), Ceftriaxone-Sulbactam (30/15 mcg), Ceftriaxone-Tazobactam (30/10 mcg), Ciprofloxacin (5 mcg), Colistin (10 mcg), Doxycycline (30 mcg), Enrofloxacin (10 mcg), Erythromycin (15 mcg), Gentamicin (10 mcg), Moxifloxacin (5mcg), Ofloxacin (5 mcg), Oxytetracycline (30 mcg), Penicillin-G (10 units), Streptomycin (10 mcg) and Tetracycline (30 mcg). The diameters of the respective zones of inhibition were measured and the results were interpreted as sensitive, intermediate or resistant to the antibiotic as per the CLSI^[16]^.

## Results

### Bacterial isolates and their resistance pattern:

**Figure 1.**
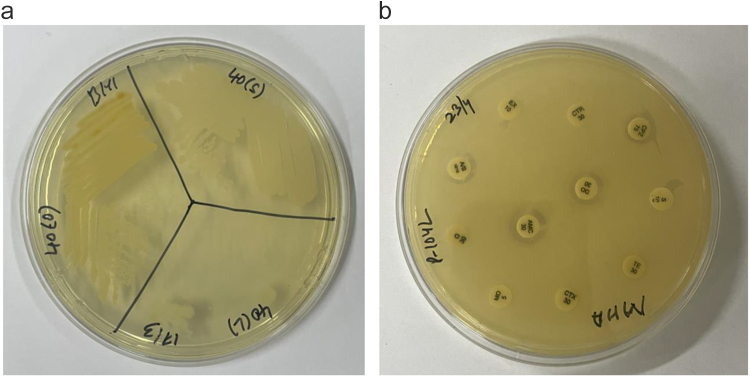
**A.** *Corynebacterium* spp. (O), *Staphylococcus* spp. (S) and *Bacillus* spp. (L) isolated from a single milk sample, **b.** A multidrug resistant isolate of *E. Coli.*

**Figure 2.**
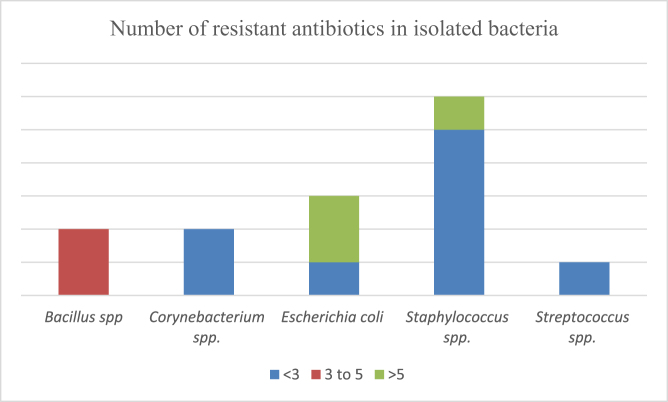
Number of resistant antibiotics against bacterial isolates.

The microbiological testing of the milk samples of goats suffering from clinical mastitis revealed the presence of *Bacillus* spp. (21.42%), *Corynebacterium* spp. (14.28%), *Escherichia coli* (21.42%), *Staphylococcus* spp. (35.71%) and *Streptococcus* spp. (7.14%). *Staphylococcus* spp., *Streptococcus* spp. and *Corynebacterium* spp. were resistant to less than three antibiotics tested. *Bacillus* species showed resistance to three to five antibiotics tested; whereas the *Escherichia coli* were highly resistant with all the isolates showing resistance to more than five antibiotics tested, except one. Figure 1a depicts the bacterial isolates *Corynebacterium* spp., *Staphylococcus* spp. and *Bacillus* spp. from a single milk sample whereas, 1b depicts a multidrug resistant isolate of *E. coli*. The number of antibiotics resistant against bacterial isolates have been compared graphically in Fig. 2.

### Overall resistance of bacteria to specific antibiotics

**Figure 3.**
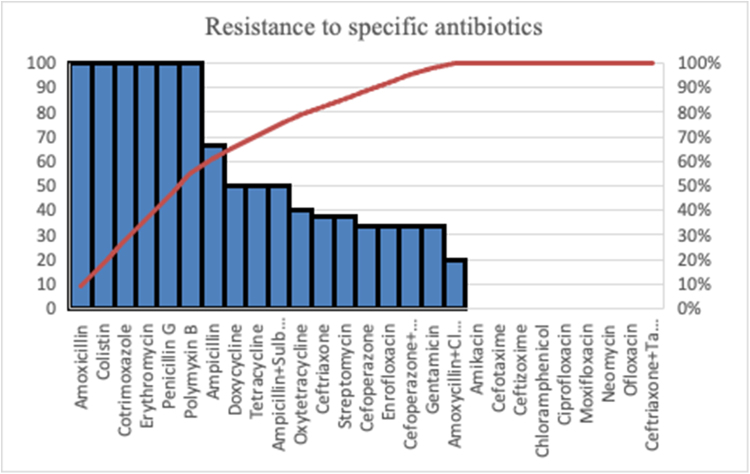
Overall resistance pattern of the bacteria to specific antibiotics.

**Figure 4.**
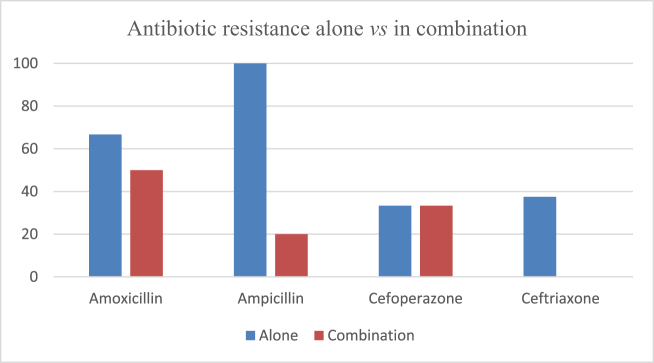
Antibiotic resistance pattern in individual use vs combination.

The antimicrobial susceptibility testing of the bacterial cultures from the milk samples revealed resistance to multiple antibiotics tested against the pathogens associated with the clinical mastitis in goats. A very high antimicrobial resistance was found in penicillin G (100%), amoxicillin (100%), erythromycin (100%), polymyxin B (100%) and colistin (100%), followed by ampicillin (66.67%), doxycycline (50%) and tetracycline (50%). Comparatively lower resistance was observed in case of oxytetracycline (40%), streptomycin (37.5%), ceftriaxone (37.5%), cefoperazone (33.33%), gentamicin (33.33%) and enrofloxacin (33.33%). The antibiotics including amikacin (0%), cefotaxime (0%), ceftizoxime (0%), ciprofloxacin (0%), moxifloxacin (0%), ofloxacin (0%) and neomycin (0%) were found to have minimum resistance in the tested isolates. The bacteria had quite low resistance against antibiotic combinations such as ceftriaxone-tazobactam (0%), amoxicillin-clavulanate (20%), cefoperazone-tazobactam (33.33%) and ampicillin-sulbactam (50%). The overall resistance pattern of the bacteria to specific antibiotics has been depicted *via* a Pareto Chart in Fig. 3 while, Fig. 4 depicts the comparison of resistance pattern in case of individual use vs combination of antibiotics.

### Multi drug resistance pattern of bacteria per sample

**Figure 5.**
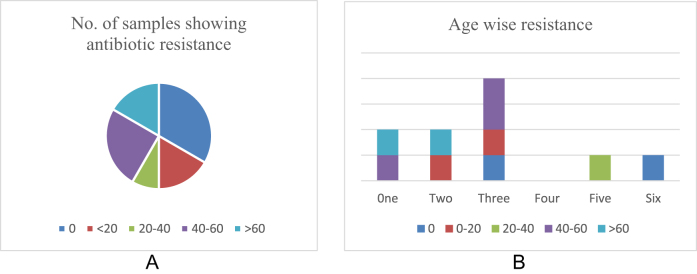
**A**. Sample wise and, **b.** Age wise multidrug resistance pattern of bacteria.

The mastitic milk samples were obtained from the goats of the age group 1.5 up to 6 years. Isolates from half of the goats had developed resistance to more than two antibiotics most of which were 3 years old. The data across the different age groups of goats were variable and not indicative of the age as a factor in the development of antibiotic resistance. Five of the processed samples had not developed any bacterial growth during incubation which may be due to the recent antibiotic usage in the animals prior to collection of the milk samples. The sample wise and age wise multidrug resistance pattern of bacteria has been depicted in Fig. 5a and b, respectively.

### Resistance of bacteria to different classes of antibiotics

**Figure 6.**
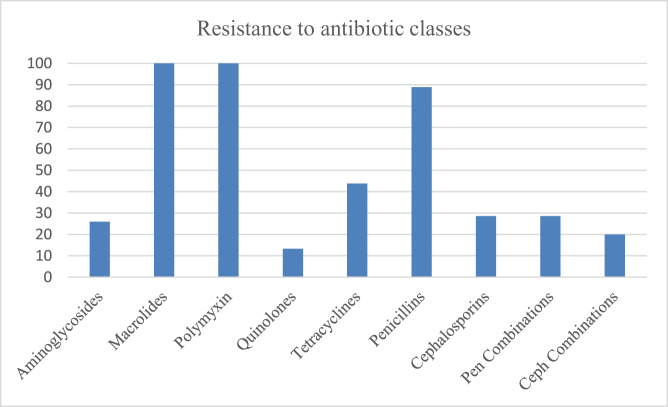
Antibiotic resistance pattern in various classes of antibiotics.

The bacterial cultures isolated from the milk of mastitic goats showed variable resistance to the various antibiotic classes. The highest antibiotic resistance was evident against macrolides (100%) and polymyxins (100%) followed by the penicillins (88.89%) and tetracycline group of antibiotics (43.75%). The resistance against the cephalosporins (28.57%), aminoglycosides (25.93%) and quinolones (13.33%) was quite lower. The resistance in case of the antibiotic combinations i.e. the penicillin combinations (28.57%) and cephalosporin combinations (20%) was comparatively lower as compared to their individual use. Figure 6 depicts the antibiotic resistance pattern observed in various classes of antibiotics.

## Discussion

Mastitis is a major challenge faced by the livestock sector today. Milking of goats is commonly done in India by hands and is not mechanized, thus the risk of mastitis is high because of poor hygiene and managemental practices. Investigation of the aetiology of mastitis may be helpful in finding the bacteria involved, the status of their sensitivity to the various antibiotics and possible ways of mitigation. Hence, the present study aimed to investigate the bacteria associated with caprine mastitis and their antimicrobial susceptibility profiling.

In the present study, an investigation of the prevalence of bacterial isolates revealed that the most commonly isolated bacteria from the caprine milk samples were *Staphylococcus* spp. (35.71%). The finding is in agreement with other studies done in goats^[17,18]^ as well as bovines^[19-22]^ which indicate that *Staphylococcus* spp. are the most common bacteria cultured from the mastitic milk. The other common bacteria found were *Bacillus* spp. (21.42%), *Corynebacterium* spp. (14.28%), *Escherichia coli* (21.42%) and *Streptococcus* spp. (7.14%). Bradley and Green^[23]^ have reported *E. coli* to be the predominant pathogen which is not completely consistent with our findings. On the other hand, Hussain *et al*^[24]^ have reported *Staphylococcus* spp. (51.4%) as the most commonly isolated pathogen. The study done by Ali *et al*^[25]^ has indicated a similar isolation profile i.e. *Staphylococcus aureus* (45.3%), *Streptococcus* spp. (22.7%), *E. coli* (11.6%) and *Klebsiella* spp. (3.7%), although *Klebsiella* spp. was not isolated in the present study and the prevalence of *E. coli* has been found to be higher as compared to the *Streptococcus* spp. Among the members of the *Enterobacteriaceae* family, the occurrence of *E. coli* has been found to be significantly higher than other Gram negative bacteria^[19]^. Other studies have also found *E. coli* to be the most common cause of coliform mastitis; similarly in the current study, *E. coli* (21.42% of total isolates) was the second most commonly isolated bacteria after the *Staphylococcus* spp. and was the only *Enterobacteriaceae* member reported.

Overall, the *Staphylococcus* spp. has been found to be the most commonly cultured mastitic pathogen in countries like Spain (70%)^[26]^, Kenya (60.3%)^[27]^ and USA (38.2%)^[28]^. In accordance with our findings, another study has also found *Bacillus* spp. in the goats suffering from subclinical mastitis at a little bit higher prevalence rate (27.1%) apart from *Staphylococcus* spp. (38.98%) and *E. coli* (10.2%)^[29]^. The management and geographical location of farms may contribute to the variations in prevalence rates of the isolated bacteria involved in caprine mastitis^[30]^. In the present study, no bacterial growth occurred in 29.41% of caprine mastitic milk samples although the mastitis causing pathogens may be detected in such culture-negative samples through PCR^[31]^. Similar rates of samples with no bacterial growth have also been reported in other studies like 28 percent culture negative samples found by Al-Harbi and co-workers^[19]^. Absence of growth in milk samples may occur due to very low concentration of the bacteria, or presence of anaerobic bacteria which are missed in aerobic incubation, or due to recent antibiotic treatment of the animal.

The isolation and identification of the bacterial isolates was followed by the antimicrobial susceptibility profiling in which the *Staphylococcus* spp. were found to be highly susceptible to most of the antibiotics tested, with none of the isolate being resistant to more than three antibiotics tested. Although the isolates were resistant to penicillin group of antibiotics including penicillin G and amoxicillin when not given in combination with clavulanic acid. This finding is in accordance with a few other studies as well^[32-34]^. The *E. coli* isolates have shown high resistance rates to the beta lactam antibiotics as well as tetracyclines and aminoglycosides, which has been demonstrated in many other studies too^[20,35]^. Hence, the beta lactam antibiotic combinations with sulbactam, tazobactam or clavulanic acid may be used for the treatment of coliform mastitis involving *Escherichia coli*. The *Streptococcus* spp. and *Corynebacterium* spp. isolated in the present study showed high susceptibility to majority of the antibiotics hence the first generation antibiotics may be used for the front-line treatment of the caprine mastitis in such cases. The *Bacillus* spp. were resistant to three to five antibiotics including penicillins and tetracyclines while, resistance to certain antibiotics belonging to the aminoglycosides, cephalosporins group, etc. was found to be variable. The pathogens could express multidrug resistance due to the continuous antibiotic exposure or genetic acquisition of resistance through the plasmids or transposons^[36]^. It has been reported by Zhao *et al*^[37]^ that antibiotics such as chloramphenicol, cephalexin, kanamycin, tetracycline and sulfamethazine, enhance the co-selection of the genes related to resistance in other antibiotic classes. Studies indicate that multidrug resistance could occur due to the combined mechanisms of multiple resistance^[38]^.

Overall, in the present study, very high antibiotic resistance was observed against the macrolides (100%) and polymyxins (100%) followed by the penicillins (88.89%) and tetracycline group of antibiotics (43.75%). However, certain studies have reported higher susceptibility to penicillins like amoxicillin which was found to be very effective by Jabbar *et al*^[18]^, which could be due to less use of this drug for the treatment of mastitis in their region of study at the time of undertaking the study. Similarly, in contrast to the lower sensitivity of tetracyclines reported in the present study, the tetracycline has been reported to be highly efficacious against caprine mastitis in certain studies^[39-41]^. Efficacy of the gentamicin against bacteria involved in caprine mastitis has been reported to be high by Ali *et al*^[25]^ which is also seen in the present study regarding other aminoglycosides including streptomycin and amikacin as well. The resistance against quinolones (13.33%) was also found to be quite lower in the present study, which is in accordance with studies where certain mastitic pathogens have been found to be highly susceptible to quinolones such as 0% resistance of ciprofloxacin reported by Kabui *et al*^[42]^.

The resistance against the antibiotic combinations such as ceftriaxone-tazobactam, amoxicillin-clavulanate, cefoperazone-tazobactam and ampicillin-sulbactam has been found to be very low. Similarly, study done by Kabui *et al*^[42]^ also revealed the high efficacy of amoxicillin-clavulanate as compared to the amoxicillin alone. The resistance against the cephalosporins was found to be quite low, and even lower in the combinations such as ceftriaxone-tazobactam and cefoperazone-tazobactam. While the third generation cephalosporins are still effective in many cases, the beta lactamase inhibitors including sulbactam and tazobactam may increase the effectiveness of the penicillins and cephalosporins in combating the resistant mastitic pathogens or beta lactamase producers. Since the interpretation criteria and methods of studying antibiotic resistance vary, the easy and efficient comparison of the different studies is difficult. Furthermore, the variation in the antimicrobial activity of different antibiotic classes emphasizes the importance of determining the antibiotic susceptibility patterns for the various pathogens associated with caprine mastitis.

## Conclusion

Proper treatment and culling policy for the clinical as well as sub-clinical mastitis is essential to limit the spread of contagious mastitis in the herd. The *in vitro* study of antimicrobial susceptibility provided insights into the development of antimicrobial resistance in goats indicative of the potential spread to human beings. The goats can also be utilized in future research for *in vivo* studies bridging the gap between the lab results and human trials. Moreover, as the antimicrobial susceptibility was found to vary across the goats suffering from mastitis as well as the bacteria involved, the bacterial isolation and antimicrobial susceptibility testing is recommended prior to initiating the antimicrobial treatment. This would be crucial in selection of the appropriate antibiotic to limit the development of antimicrobial resistance in goats and its spread to humans as well. Further research should be conducted for elucidating the genetic basis of antibiotic resistance in the goats of study area so that effective policy decisions may be taken for the control of multidrug resistant isolates.

## Data Availability

Data can be obtained from the corresponding author on reasonable request.

## Animal welfare statement

The study involved collection of the milk samples humanely from the animals, demonstrating a high standard of veterinary care and with prior informed consent of the animal owner.
